# Exploring the relationship between migraine and mental health – perspectives from the patient and physician experience

**DOI:** 10.3389/fneur.2026.1773577

**Published:** 2026-04-01

**Authors:** Jaclyn E. Klepper, Heather Moran, Lawrence Newman

**Affiliations:** 1 Pfizer, Inc., New York, NY, United States; 2 American Migraine Foundation, New York, NY, United States; 3 Atria Global Health and Research Institute, New York, NY, United States

**Keywords:** anxiety, clinician-patient dialogue, depression, mental health, migraine

## Abstract

**Objective:**

There is growing interest in the impact of multimorbidity in migraine, especially between migraine and mental health conditions. The relationship between migraine and mental health is bi-directional, people with migraine are more likely to experience anxiety and depression and people with anxiety and depression are more likely to experience migraine. Despite advancements in migraine treatment, there is limited awareness and access to mental health treatment for patients with migraine. This study aimed to examine the relationship between migraine and mental health and explore the perspectives of patients living with migraine and physicians treating migraine.

**Methods:**

A cross-sectional, observational, online survey conducted between April and May 2022 to describe the experience and perspectives of patients living with migraine, and the physicians treating migraine.

**Results:**

1,100 people (84.7% female; mean age, 46.4 years) with self-reported migraine and 302 physicians who treat migraine completed the survey. Nearly half of patients with migraine (48.8%) reported their mental health as” poor” or “fair,” with 78.9% endorsing an anxiety disorder and 63.6% depression. Most patients (84.1%) and physicians (88.7%) agreed that as migraine attacks worsen or become more frequent, mental health burden increases. People with migraine reported feelings of frustration (45.1%), stress (37.0%), and anxiety (34.2%), whereas physicians reported those feelings in 70.9%, 56.3%, and 37.4% of their patients, respectively. Only 28.1% of people with migraine stated their physician frequently asked about their mental health, whereas 70.5% of physicians stated they often do so. Half (49.0%) of patients were not satisfied with the mental health guidance they receive from the physician treating their migraine. Most of both groups (patients, 87.0%; physicians, 93.7%) agreed that improved migraine control would benefit patients’ mental health.

**Conclusion:**

This study emphasized the importance of optimizing the management of migraine and mental health needs of patients concurrently. Physicians who treat migraine should be sensitive to mental health comorbidities, and internal and external stigmas experienced by patients. There is a need for providing physicians who treat migraine with additional educational initiatives and resources geared toward understanding patient needs, as well as having comprehensive care plans and resources available to provide support to patients.

## 1 Introduction

Migraine is a highly common neurologic disorder affecting nearly 1 billion individuals globally, with an estimated 1-year prevalence of approximately 11.7%–14.7% in the United States (US) (1, 2). Neurological disorders are the leading cause of disability and lost years of healthy life combined, with migraine being the second most frequent neurological cause of disability in the world (3).

Multimorbidity refers to people living with two or more (chronic) health conditions and has been reported to be rising on a worldwide scale (4). Consequences of multimorbidity include reduced quality of life (physical and mental components), higher disability, increased healthcare costs and utilization, and increased mortality (5–8). Within the past decade, there has been greater interest in researching the impact of multimorbidity in migraine, with a specific focus on the association of migraine with mental health conditions. A longitudinal analysis of over 50,000 people demonstrated that physical health conditions, including migraine, were often clustered with mood and anxiety (9). Within large population-based studies, the bi-directional association between migraine and mental health has been established, with individuals with migraine being 60%–80% more likely to develop depression and those with depression being almost twice as likely to develop migraine (10, 11). Similarly, migraine and anxiety disorders each increase the risk of developing the other, with more than 50% of patients with migraine meeting the criteria for an anxiety disorder within their lifetime (12). These findings were supported by earlier studies linking mental health concerns and migraine (13–15) and may be partly attributable to shared underlying physiological mechanisms (16, 17). Furthermore, the American Headache Society’s consensus statement definition of “success in migraine prevention” includes “improvements in health-related quality of life and reduction in psychological distress due to migraine” (18). Despite this acknowledgement, there are a limited number of measures specific to patients with migraine in distinguishing mental health from the general burden of migraine. Therefore, it is imperative to consider migraine and mental health independently and together. Although screening tools, such as the Patient Health Questionnaire (a brief self-report consisting of a 2-item depression scale and a 2-item anxiety scale) and the Hospital Anxiety and Depression Scale (consisting of 2 subscales to measure anxiety and depressive disorders), have been assessed in people with migraine and performed reasonably well in controlled settings, these and similar tools have had limited uptake within clinical practice. These tools also do not account for how anxiety and depression present specifically in the context of migraine.

Migraine-related stigma is common in clinical and population-based samples and refers to the negative attitudes and beliefs related to the symptoms and characteristics of migraine. The stigma is often two-fold; external stigmas are projected onto them by the general public, friends, family, and medical professionals, and internalized stigmas refer to the negative beliefs and feelings of shame, guilt, depression, anxiety, and low self-worth (19). Stigmas in medical facilities have been shown to undermine diagnosis, treatment, and successful health outcomes (20). When stigmas arise from physicians, people may be subjected to denial or provision of sub-standard care, verbal abuse, or longer wait times for appropriate care (20). This combination of internal and external stigmas can profoundly affect an individual’s ability to care for themselves, the ability to access appropriate migraine care and a physician’s ability to care for their migraine patients (19).

“Double stigma” describes the additive effects of having two stigmatized diagnoses at the same time (21). In people with epilepsy and mental illness (felt stigma and depression), the conditions act as bi-directional risk factors and increase social anxiety, with negative impacts on relationships (being unmarried), and greater risks of unemployment (22). Although there are no definitive studies regarding the impact of double stigma in migraine and mental health, it is reasonable that similar hypotheses could be made. Epilepsy and migraine are chronic neurological disorders with episodic manifestation, characterized by recurrent attacks (23). Compared with the physical manifestation of epilepsy, the invisibility of migraine and mental health disorders are associated with higher degrees of internal and external stigmas (19). Recently, the OVERCOME Study (ObserVational survey of the Epidemiology, tReatment, and Care Of MigrainE) found that almost one-third (31.7%) of individuals with active migraine reported experiencing stigmas often or very often (24). Experiencing stigmas was associated with greater disability, reduced quality of life, and greater disease burden between migraine attacks (interictal burden). For those experiencing stigmas often or very often, interictal burden was characterized as “severe” for 80% of patients (24).

Despite treatment advancements for acute and preventive migraine management, developments focused on the treatment of mental health in patients with migraine have been less widely adopted, arising from deficits in knowledge, stigma, patient perceptions, and systemic and relational barriers (25). Although the recognition of the bi-directional impact of migraine and mental health is improving and concurrent treatment of both is supported through growing research, further research is needed to identify barriers to the appropriate mental health care of patients with migraine, specifically arising from their interactions with their attending physician. Therefore, this study aimed to further examine the relationship between migraine and mental health and explore the perspectives of physicians treating migraine and patients living with migraine on this relationship.

## 2 Methods

A cross-sectional, observational, online survey was conducted to characterize the current experience and perspectives of patients living with migraine and physicians who treat patients with migraine. Separate instruments were developed for each respondent group to reflect their unique perspectives.

Surveys were developed by Biohaven Pharmaceuticals in collaboration with the American Migraine Foundation. The content for the patient survey was informed, in part, by input from six migraine patients (members of the American Migraine Foundation) who participated in a 2-h moderated focus group. The topics discussed during the focus group were centered on patients’ experiences with living with migraine and the importance of mental health. The content for the physician survey was developed based on a review of the literature, including existing measures and expert input. Survey questions for both instruments were original in nature and designed to elicit feedback regarding respondents’ overall experiences with migraine and mental health from the patient and physician perspectives. Each instrument comprised approximately 35 questions.

Survey materials were reviewed by Advarra, an independent institutional review board, and a waiver was granted prior to study initiation (Pro00061689). The survey was conducted from April 20 to May 31, 2022. Pfizer Medical Affairs assumed responsibility for the resulting publication following acquisition of Biohaven Pharmaceuticals (October 2022).

Participants were primarily recruited in collaboration with the American Migraine Foundation, using online recruitment strategies and direct engagement of the foundation’s membership list, supplemented with a national panel of patients with migraine. Patient participants were eligible if they were US residents at least 18 years of age and had self-reported a clinician diagnosis of migraine within the previous 2 years. Additionally, patients were required to have been diagnosed and treated by a physician for a mental health condition or had self-identified as having depression, anxiety, or another mental health condition (not necessarily diagnosed or treated by a physician), as self-reported.

Physicians were recruited from a national provider database and included primary care physicians, neurologists, and headache specialist physicians who care for patients with migraine. Participating physicians were required to be board certified, have been in practice for longer than 2 years, spend a minimum of 80% of their professional time providing direct patient care, and treat at least five patients with migraine per week. No *a priori* parameters were used as quotas for recruitment.

All participants reviewed an online consent form and provided consent prior to entering the survey. Participants completed the survey online via a secure web portal/electronic data capture system that took approximately 10 min to complete. Participants were offered the incentive of the chance to win a $100 Amazon gift card after completion of the survey. All screening and survey information was self-reported.

Demographic and migraine characteristics were collected via survey items. Participants were provided with lists of characteristics and descriptions and asked to select the ones that best described their current experience or specific trait.

The surveys were by invitation only and administered via Confirmit, an online survey-hosting platform, and consisted of multiple-choice questions, rank-order questions (order of importance 1–5), and scale-based responses using a symmetrical 5-point Likert scale of agreement with presented statements (from “strongly disagree” to “strongly agree”), consistent with established self-reporting survey methodology (26). A small number of questions used adaptive questioning. All questions were close ended.

No statistical power calculation was conducted prior to the study. For descriptive analyses, continuous variables were summarized using means, standard deviations, medians, quartiles, and minimum and maximum values, as appropriate. Categorical variables were summarized using frequencies and percentages. Statistical testing was two tailed and considered significant at *p* < 0.05. Categorical variables were analyzed using x^2^ test or Fisher’s exact test (if counts were <5) and continuous variables were analyzed using independent-samples t-test or Mann Whitney U (if non-normal). Statistical analyses were conducted in Excel (Microsoft 2025, version 2,511).

## 3 Results

The survey was completed by 1,100 people with self-reported migraine and 302 physicians who treat patients with migraine between April and May 2022. Most patient participants were female (84.7%) and White (88.5%), and mean age was 46.4 years (range, 18–86 years) (Table 1). Patients reported a mean of 19.1 years from initial migraine diagnosis and that they had experienced a mean of 14.8 headache days and 10.0 migraine days within the past month.

**Table 1:** Patient respondent demographics and migraine characteristics.

Characteristics	All respondents *N* = 1,100
Age, mean ± SD (years)	46.4 ± 13.7
Gender, *n* (%)	
Female	932 (84.7)
Male	149 (13.5)
Non-binary	14 (1.3)
Transgender	3 (0.3)
Prefer not to respond	2 (0.2)
Ethnicity, *n* (%)	
White	973 (88.5)
Hispanic, Latino, or Spanish origin	85 (7.7)
Black	62 (5.6)
Asian	20 (1.8)
American Indian	20 (1.8)
Alaskan Native	1 (0.1)
Middle Eastern	5 (0.5)
North African	3 (0.3)
Native Hawaiian	4 (0.4)
Pacific Islander	5 (0.5)
Other or prefer not to respond	16 (1.5)
Time from migraine diagnosis, mean ± SD (years)	19.1 ± 14.3
Number of headache days per month, mean ± SD (days)	14.8 ± 8.7
Number of migraine days per month, mean ± SD (days)	10 ± 7.7
SD, standard deviation.	

Of the 302 physicians included, most were primary care physicians (66.9%), followed by neurologists (16.6%) and headache specialists (16.6%). Physicians had been in practice for a mean ± standard deviation of 19.7 ± 8.5 years, dedicated 95.2% (mean) of their professional time to direct patient care, and treated a mean ± standard deviation of 36.7 ± 40.2 patients with migraine each week (Table 2). Of 51 states/districts within the US, 82.3% (*n* = 42) were represented.

**Table 2:** Physician respondent demographics and characteristics.

Characteristics	All respondents *N* = 302
Specialty, *n* (%)	
Neurologist	100 (33.1)
Neurology subspecialty, “yes,” *n* (%)	68 (68.0)
Headache medicine	46 (67.8)
Pain medicine	4 (5.9)
Epilepsy	3 (4.4)
Movement disorder	2 (2.9)
Vascular neurology	1 (1.5)
Clinical neurophysiology	4 (5.9)
Other	8 (11.8)
Primary care/family medicine/internal medicine	198 (65.6)
Pain medicine	4 (1.3)
Practice setting, *n* (%)	
Community-based solo private practice	42 (13.9)
Community-based group private practice	183 (60.6)
Academic or teaching hospital	46 (15.2)
Community non-teaching hospital	19 (6.3)
Private non-teaching hospital	4 (1.3)
Government/VA hospital	1 (0.3)
Tertiary care center	1 (0.3)
Other	6 (2.0)
Proportion of time spent in direct patient care, mean ± SD (%)	95.2 ± 6.3
Number of patients treated per week, mean ± SD (*n*)	36.7 ± 40.2

SD, standard deviation; VA, veterans affairs.

### 3.1 Prevalence of mental health difficulties

**Figure 1:**
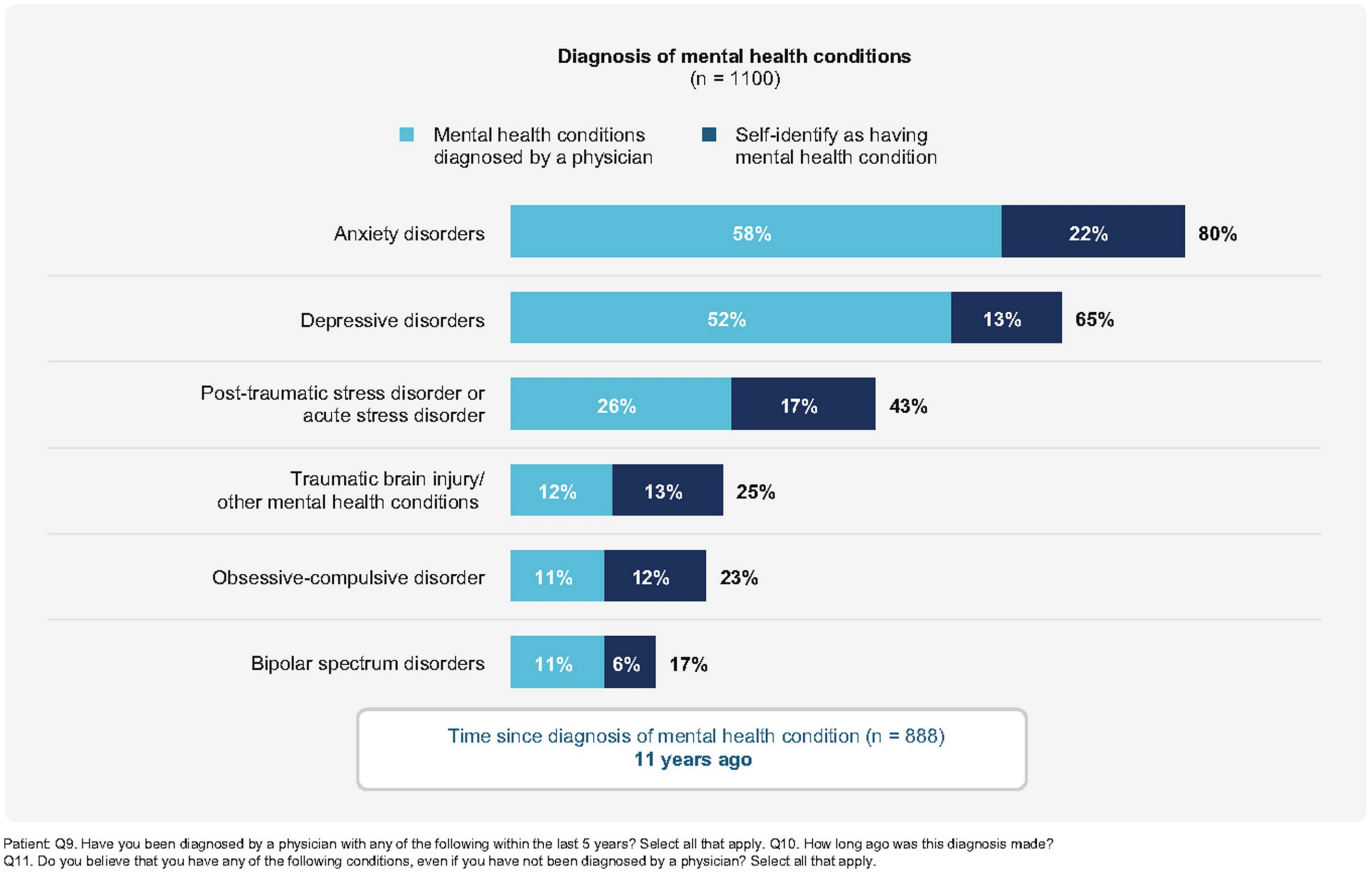
Patient diagnosis of comorbid mental health conditions (*n* = 1,100).

When patients were asked to rate their current mental health using a scale of “poor,” “fair,” “good,” or “excellent,” nearly half of patients (48.8%) with migraine self-reported that they felt as though their mental health was “poor”(8.8%) or “fair” (40.0%). When presented with a list of mental health disorders and asked to select all that apply, the most commonly identified disorders were anxiety disorders (78.9%; 57.5% diagnosed by a physician; 21.8% self-reported by patient) and depression (63.6%; 51.5% diagnosed by a physician; 12.6% self-reported by patient), either diagnosed by a physician or self-described (Figure 1).

### 3.2 Relationship between migraine and mental health

Results demonstrated that most respondents, including patients with migraine and physicians who treat migraine, recognized and acknowledged that migraine and mental health are related and impact each other. Notably, 88.7% of physicians and 84.1% of patients agreed (*p* = 0.0006) that as migraine attacks worsen or become more frequent, mental health burden increases. The inverse also holds true; 91.7% of physicians and 65.5% of patients agreed (*p* < 0.0001) that as mental health issues become more severe, migraine burden increases. Nearly all physicians (92.4%) expressed that migraine and comorbid mental health symptoms may negatively impact a patient’s ability to successfully manage their migraine and adhere to their management plan.

Patients with migraine (86.0%) and physicians (88.7%) acknowledged that uncertainty around when the next migraine will occur is a key contributor to reported worry and anxiety, perpetuating a cycle of worry, anxiety, and migraine attacks.

**Figure 2:**
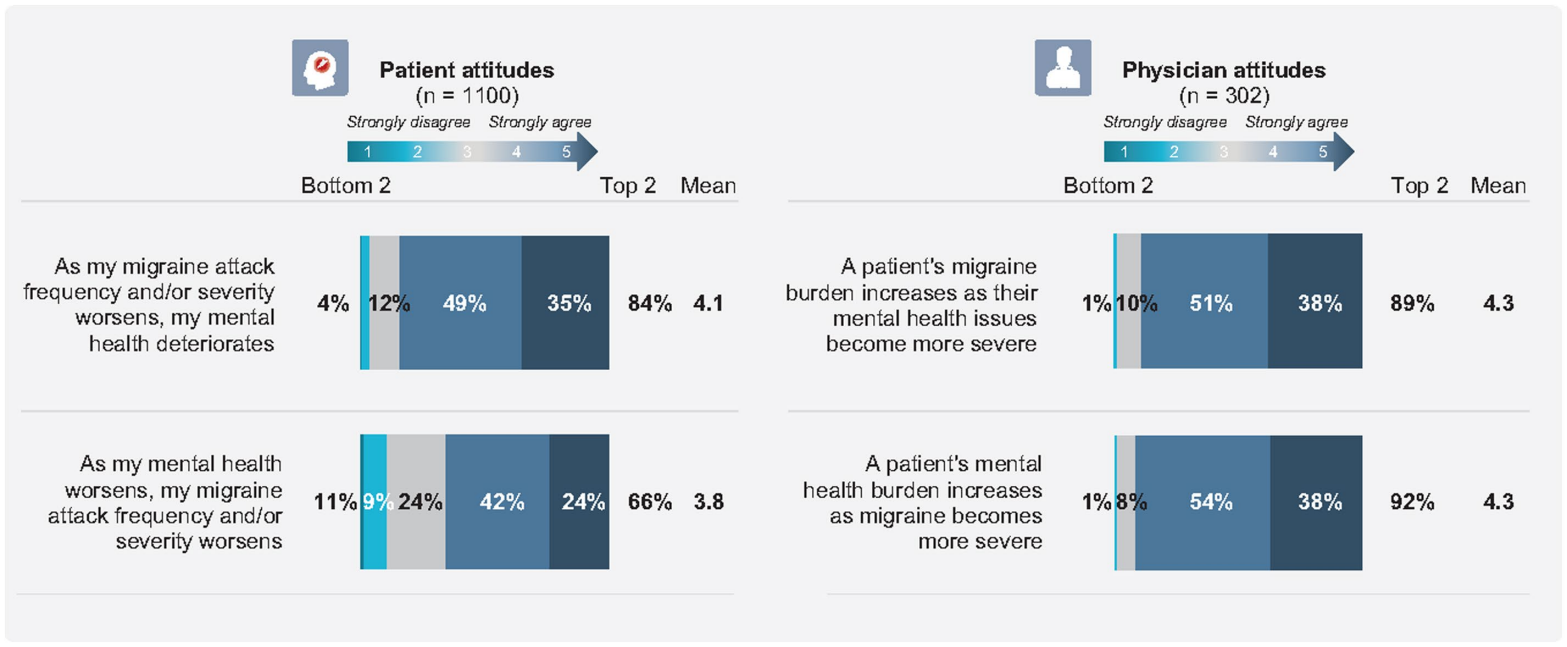
Attitudes about migraine and mental health.

Attitudes about migraine and mental health, as reported by patients and as perceived by physicians, are summarized in Figure 2.

**Figure 3:**
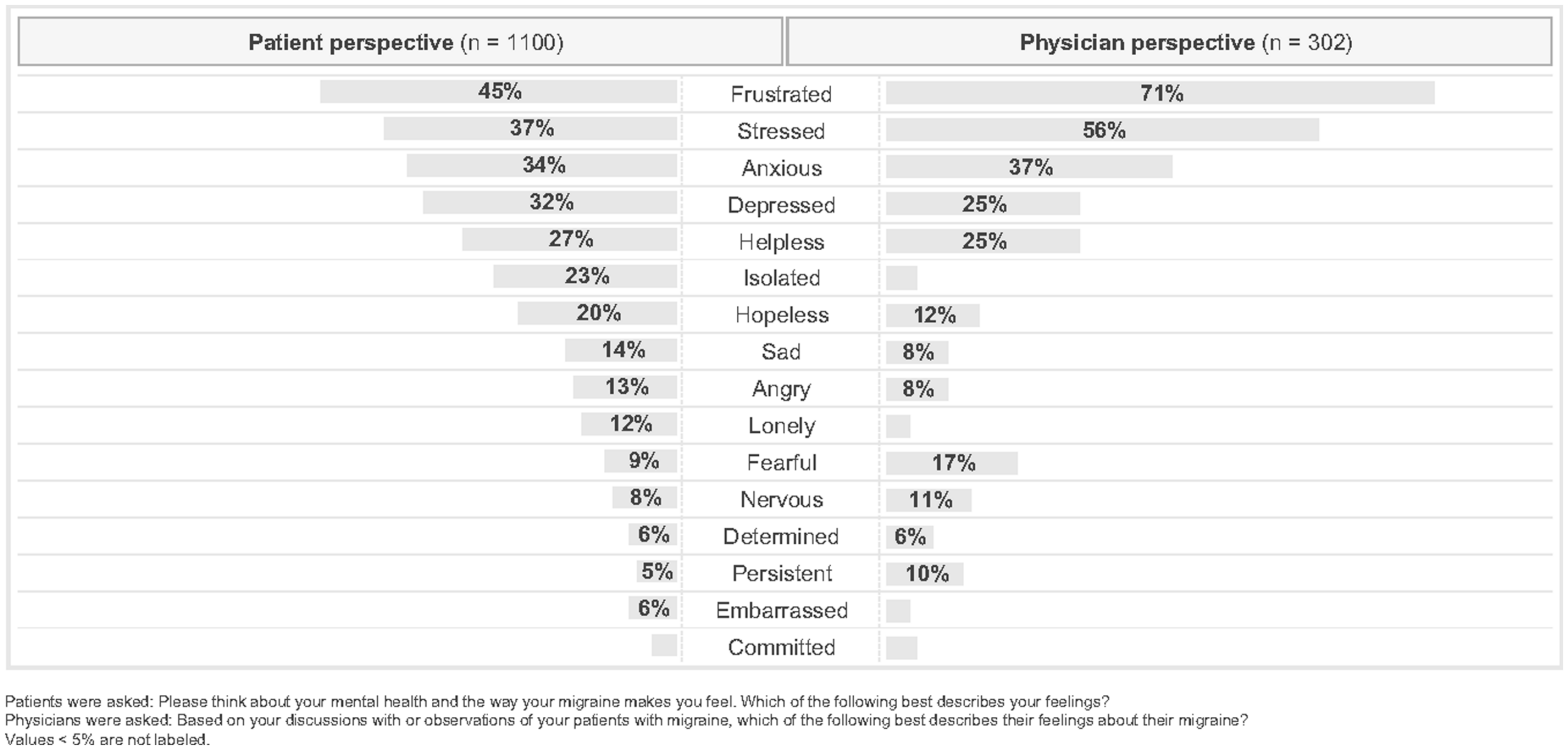
Feelings and emotions about migraine as reported by patients and perceived by physicians.

When patients were asked to think about their mental health and the way their migraine makes them feel, they responded that they experience stress (37.0%) and frustration (45.1%) as a result of their migraine attacks. In addition, approximately one-quarter of patients also noted that they feel a sense of isolation (22.9%) and helplessness (26.5%) (Figure 3). Physicians were asked to reflect on discussions with or observations of their patients with migraine and describe how they believe their patients feel about their migraine. Interestingly, despite 20.3% of patients reporting feelings of hopelessness and 22.9% of patients reporting feelings of isolation, physicians failed to recognize these to the same degree, reporting 11.8% and 4.0%, respectively. Statistical comparison of the responses provided by patients and physicians were significantly different for feelings of hopelessness (*p* = 0.0013), isolation (*p* = 0.0001), loneliness (*p* = 0.0001), and depression (*p* = 0.0224) (Figure 3). Feelings of anxiousness were recognized at similar levels between patients (34.2%) and physicians (37.4%).

### 3.3 Physician-patient dialogue

Although the inter-relationship between migraine and mental health is well recognized by patients and physicians, this subject may not be adequately discussed during clinical visits. Nearly two-thirds (61.5%) of patients with migraine felt that it was very to extremely important to discuss mental health with the physician treating their migraine. Despite feeling that mental health is an important topic, only 40.7% of patients recalled their physician explaining the existence of a connection between migraine and mental health. Further, only about one-quarter (28.1%) of patients with migraine stated their physician frequently asked them how migraine impacted their mental health (stress, depression, and anxiety). This is in contrast with the 70.5% of physicians who stated they often asked their patients about their mental health. However, physicians (57.6%) did acknowledge that they are often limited by insufficient time during appointments to address mental health issues.

**Figure 4:**
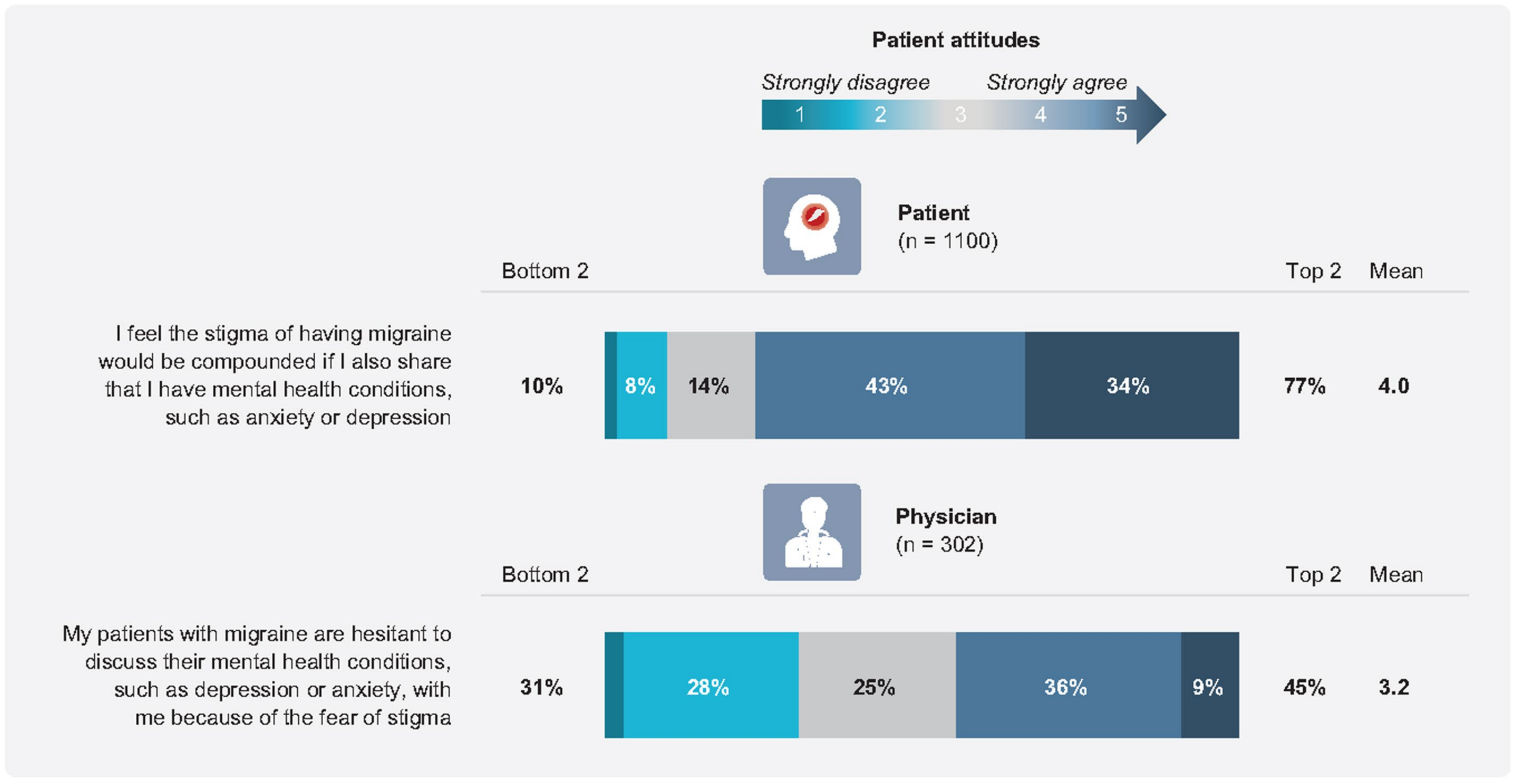
Attitudes associated with stigma and migraine.

Patients expressed reservations about initiating discussions about mental health with their physicians, noting concerns that their physicians treating their migraines viewed their mental health as a separate issue (49.2%). Physicians were somewhat unaware of patient hesitancy in discussing the impact of migraine on their mental health. One-third (32.5%) of physicians reported that they got the impression from their patients with migraine that they would prefer that appointment time be focused primarily on managing their migraine attacks. Despite wanting to discuss their mental health with the physician treating their migraine, patients with migraine experienced stigmas associated with their condition, and most agreed these stigmas would be compounded by sharing about their mental health issues (76.7%). In contrast, only 44.7% of physicians agreed that patients with migraine were hesitant to discuss mental health with them due to perceived stigmas, a statistically significant difference (*p* < 0.0001) (Figure 4).

### 3.4 Mental health treatment

Many (76.2%) physicians reported that they feel it is their responsibility to discuss mental health with their patients with migraine, and 87.7% agree or strongly agree that it would be beneficial to patients if migraine and mental health were treated concurrently. However, 40.7% of physicians reported that they did not feel that it is primarily their responsibility to treat their patients’ mental health issues as they relate to migraine. Not surprisingly, half of patients (49.0%) were not satisfied with the care they were receiving from their migraine physician for their mental health. This result differed depending on the physician’s specialty, with patients under the care of neurologists being the least satisfied (59.8%), followed by those under the care of primary care physicians (43.7%) and headache specialists (39.4%).

Nearly all (85.4%) physicians reported that they have access to experienced psychotherapists and have the ability to directly refer patients. When asked if they had ever sought care from another physician to treat their mental health, 81.2% of patients reported that they have sought care from another provider as it pertains to their mental health. This contrasts with the one-third (36.4%) of patients with migraine who reported that they have been referred to another provider for mental health treatment.

Patients with migraine and treating physicians perceived mental health treatment interventions for people with migraine to be beneficial. Treatments included prescription medication, mindfulness training, and psychotherapy/cognitive behavioral therapy. Among presented treatment options, the extent of the perceived benefit varied between patients and physicians. More physicians than patients, 77.5% vs. 58.2%, respectively, had observed benefits of prescription medication in treating mental health. The same held true for the benefits of psychotherapy/cognitive behavioral therapy, with 72.2% of physicians noting a treatment benefit compared with 54.4% of patients (*p* < 0.0001).

### 3.5 Improved migraine treatment

**Figure 5:**
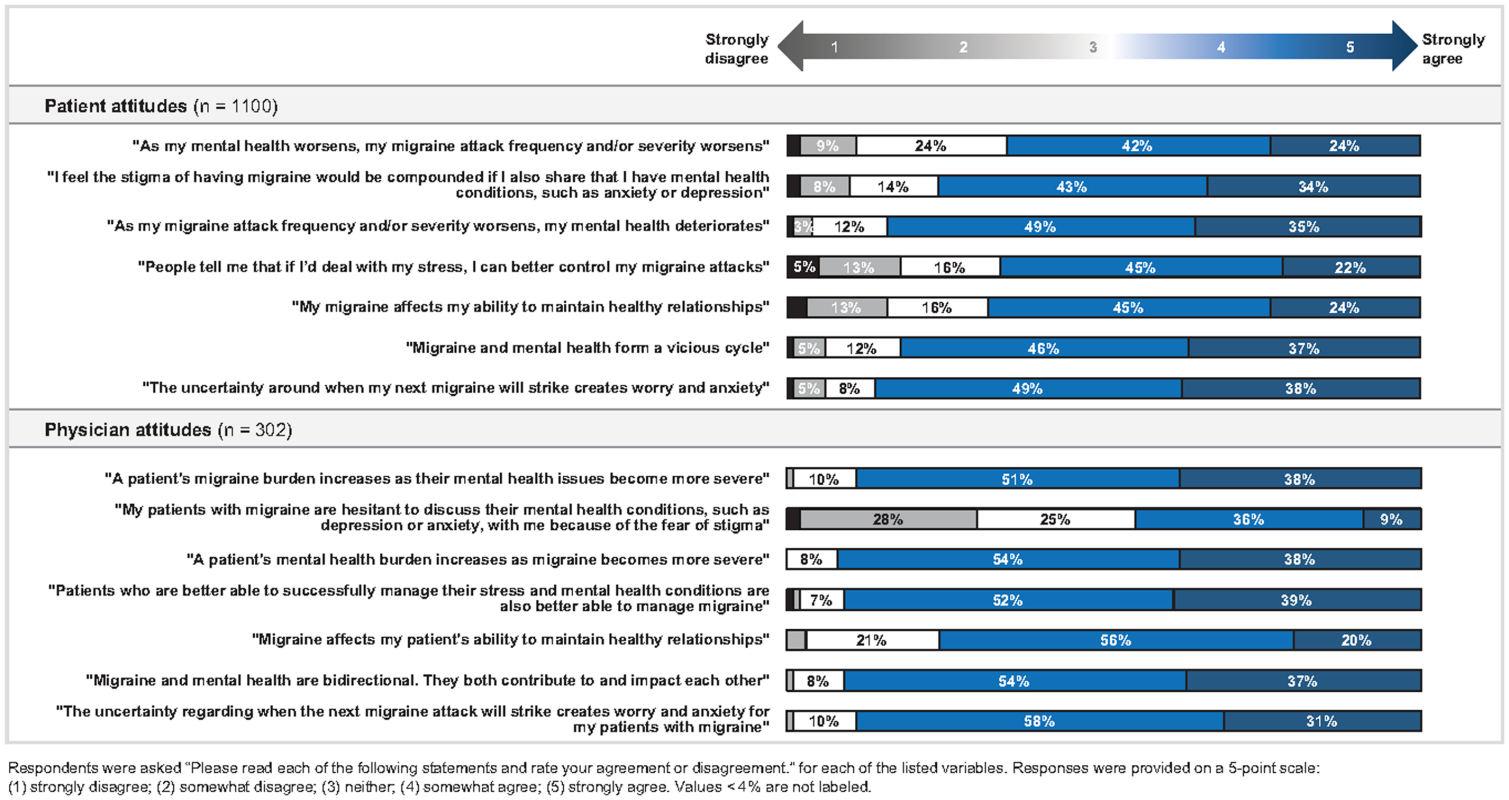
Patient and physician statements associated with migraine treatment.

The majority of patients with migraine (87.0%) and most physicians (93.7%) agreed or strongly agreed that patients’ mental health would benefit from improved migraine control (*p* = 0.0007). Nearly all physicians (91.4%) and the majority of patients (53.7%) agreed or strongly agreed that migraine management needs to be more flexible by tailoring treatment to patient needs (*p* < 0.0001) (Figure 5).

## 4 Discussion

This survey represents the first large-scale study of patients with migraine and physicians who treat patients with migraine to explore patients’ and physicians’ concurrent perspectives and experiences with migraine and mental health. The aim of the survey was to provide a more comprehensive characterization of migraine and mental health to inform clinical management and further research. Results demonstrated that, overall, nearly half of those with migraine felt as though their mental health was” poor” or “fair.” Anxiety and depression disorders were common, with 58 and 52%, respectively, being diagnosed by a physician. This is relatively high compared with other studies. In a study of over 6,000 US patients with migraine, 18.7% were reported as having anxiety symptoms, 6.1% as having depressive symptoms, and 23.8% as having both (27). However, symptoms of anxiety and depression are more common in patients with chronic migraine compared with episodic migraine and may partly account for the observed difference. Patients and physicians agreed that migraine and mental health have a bi-directional impact; more specifically, migraine attacks, inclusive of the attack itself and the potential anxiety and worry during the interictal phase, negatively impact mental health. Conversely, patients with mental health concerns may experience difficulties with migraine management, resulting in poor disease-related outcomes (28). As migraine, anxiety, and depressive disorders were frequently reported comorbidities, it was not surprising that most respondents felt that it was important for mental health to be part of the patient–physician dialogue. There was a disconnect, however, as physicians felt that they were frequently informing and having discussions with their patients with migraine about the relationship between migraine and mental health, whereas patients did not recall having these discussions as frequently and expressed a preference for the physician to initiate the conversation. This finding reinforces that even though physicians reported they are talking to their patients about mental health, patients did not recall these discussions, signaling the need for more effective and frequent communication with patients about their mental health and for patients to recognize that having mental health discussions with a migraine physician is appropriate. This may stem from how physicians are engaging with their patients. A study of 20 neurologists from the US reported that most neurologist–chronic migraine patient dialogues did not use standard communication techniques, such as open-ended questions or “ask-tell-ask” (29). A movement toward a higher degree of patient participation by encouraging shared decision-making is considered to lead to greater participation in wellness programs (e.g., regular exercise and rest, good nutrition, and avoidance of headache triggers) and, consequently, better control of the disorder (30).

Although most physicians reported having access to appropriately trained mental health professionals to refer their patients with migraine to, far fewer patients themselves reported ever receiving such a referral. Although the proportion of referrals made or not made to trained psychotherapists or the ability of patients to access this care was not determined, it was suspected that patient referrals to professionals with specialization in migraine and mental health may be hampered by the limited number of these professionals available. Although this may reflect a lack of sufficient funding, especially in regard to mental health, there is also need for greater coordination of care between physicians and mental health providers (31). In the absence of a referral, patients should be encouraged to talk to their primary care provider or attend a specialist mental health clinic. Further information, support, and resources can be found through advocacy groups.

The majority of patients and physicians were in agreement that improved migraine control would benefit a patient’s mental health. Although there are treatments available that may improve migraine, patients and physicians also tended to agree that there were limitations in current migraine treatment. For example, non-specific migraine medications, such as topiramate, may impact mood and increase the risk of psychiatric disorders (32, 33). Psychological factors also impact outcome; depression, anxiety, and type C personality disorders (avoidant, dependent, and obsessive-compulsive) may all negatively impact efficacy (34–36). Aligning with the American Headache Society’s inclusion of anti-depressants in the migraine management algorithm, tricyclic antidepressants are among the most common medications identified as having a preventive benefit for migraine and are also used to treat various anxiety disorders (18, 37). Currently, the tricyclic antidepressant, amitriptyline, has demonstrated the best evidence for migraine prevention, and there is growing interest in serotonin-norepinephrine reuptake inhibitors, indicated for depression as an off-label preventive treatment for migraine (38). Evidence suggests that patients may also benefit from the decreased medication burden by having a single medication to treat depression and migraine; however, specific migraine treatments should not be excluded (39).

Looking beyond medications, identified opportunities for improvement included removing barriers to diagnosis and disease awareness for migraine and mental health conditions, recognizing their co-occurrence, and tailoring treatment to individuals. The suggested elements of a holistic migraine management plan should include pharmacologic agents for prevention and management of acute events, as well as adjunct treatment of symptoms associated with migraine, such as nausea, in addition to mental health treatments, including lifestyle modification, behavioral modalities, and psychotherapy to support living with a chronic disabling disorder. One example of a holistic approach to fostering a robust mental health is SEEDs (Sleep, Exercise, Education, Diet, Social support), which aims to stimulate natural physiological responses to reduce anxiety and depression and improve psychological well-being. Non-pharmacological interventions have the potential to simultaneously address migraine and psychological distress. In a systematic review to evaluate the impact of how lifestyle factors may affect migraine frequency in adults with episodic migraine, there was consistent evidence to support the implementation of lifestyle modifications (specific diets, dietary supplements, physical exercise) to aid migraine management (40). In addition, there was evidence to support that mindfulness and psychotherapeutic approaches may reduce both the frequency of migraine attacks and the overall burden of migraine. In a separate systematic review and meta-analysis, mindfulness-based interventions were associated with reductions in anxiety and depression for up to 6 months post-intervention in adults with cancer (41). Taken together, there is basis to suggest that integrated, non-pharmacological strategies may help improve management of the complex interplay between migraine severity and mental health. Furthermore, connecting patients with advocacy or support groups available within the larger migraine community can serve as an important resource and help address feelings of isolation, frustration, and stigma. In this study, although physicians reported seeing benefits of psychotherapy/cognitive behavioral therapy (to a greater extent than patients), it is not known to what extent providers were knowledgeable of these interventions, including cognitive behavioral therapy. This may also have contributed to the observed variation in how satisfied patients were with the mental health care they were receiving from their physicians. Patients under the care of a neurologist reported being the least satisfied, and those under the care of a headache specialist reported being most satisfied. This may also stem from the different nature of the appointments, as well as the training and education of each respective speciality.

In this study, patients were concerned about perceived stigma, leading to over three-quarters of patients being hesitant to discuss their mental health with their physicians. This was in contrast to physicians, with less than half agreeing that patients with migraine are hesitant to discuss mental health with them due to perceived stigma. The internal and external perceived stigmas experienced by patients with migraine can be compounded by mental health issues. The disconnect may be the result of misaligned expectations regarding who holds the responsibility for initiating these mental health– and stigma-related discussions. However, given the reluctance of patients to initiate conversations with their physicians regarding their mental health, it could be argued that physicians must be proactive in initiating discussions to ensure optimal care is provided. Efforts to reduce stigmas associated with mental health and migraine are essential and require a systematic, strategic approach. Educational campaigns can help to reduce public stigmas by reshaping societal perceptions and challenging stereotypes (42). Contact with individuals from the stigmatized group are likely to lessen prejudice, and social activism can help reduce harmful representations of the stigmatized group (42). Other strategies include reducing structural stigmas by focusing on systemic barriers, such as inadequate policies, as well as reducing internalized stigmas by providing support networks and counseling services, often via advocacy groups (42). Results from this study support the notion that further education is needed for physicians and patients to elicit discussions of mental health and to reassure patients that physicians can provide mental health treatment and advocacy.

The current findings should be interpreted considering the following limitations. The methodology of survey-based research and maintaining anonymity of participants is subject to recall bias and also prevents confirmation of self-reported information, specifically the classification or diagnosis of migraine and/or mental health disorders from diagnostic testing or medical records. Nevertheless, the perceived experience of patients is also of considerable value. This study is subject to sampling bias as participation was voluntary, required internet access, and was only administered in English; therefore, the findings may not be wholly representative of the general population. Furthermore, individuals who respond to a migraine-specific solicitation or who are members of migraine advocacy groups may have systematically different characteristics (e.g., more severe migraine, higher engagement in healthcare) than the general population. Still, the experience of this population is notable as it may reflect those who need more support and shows that even with higher engagement in healthcare, they are still dissatisfied. Survey questions were original in nature, and although questions were customized to the study objectives and evaluated through pre-testing the survey instrument, the survey itself was not derived from existing validated instruments. In addition, self-reported data are subjective in nature; however, they help provide the patients’ and physicians’ experiences, which are critical in understanding where gaps may be in the comprehensive care for people with migraine. Because both exposures and outcomes were evaluated simultaneously in the study, it was challenging to determine the directionality or temporality of the observed associations or to ascertain whether migraine influences mental health, mental health influences migraine, or if both conditions might be influenced by other confounding factors. Although most physicians were primary care physicians, with only 17% being neurologists or headache specialists, and because these results may be skewed toward primary care perspectives, these data are important as most patients will see a primary care physician first. The generalizability of these results is somewhat limited by the lack of stratification based on migraine type (chronic vs. episodic) and multivariate analysis. Finally, there was a relatively low proportion of patients from non-White ethnic backgrounds. To enhance validity of the findings of this study, similar surveys could be conducted, including outside of the US, where access to appropriately trained mental health professionals may be limited.

It is also important to recognize that the complexity of migraine has made effective assessment and definition of associated mental health burden challenging. The accurate diagnosis of migraine involves distinguishing it from other types of headaches and neurological conditions and carefully considering various factors and symptoms that may overlap with other disorders, which may be experienced and described differently by the individual. There are assessments and questionnaires that are commonly used in research to measure the impact of headache on quality of life and ability to function (Headache Impact Test, Migraine Disability Assessment questionnaire, and Migraine-Specific Questionnaire); however, the domains assessed do not fully capture the spectrum of symptoms and difficulties associated with migraine, particularly as they relate to mental health. There is variability in the individual’s experience with their symptoms, type of migraine, and ictal phase at the time of assessment. The inherent complexity of the relationship between migraine burden, associated mental health, disability, and quality of life often contributes to indistinguishable definitions.

## 5 Conclusion

The bi-directional association between migraine and mental health is complex and influences the overall patient experience as it relates to their clinical course, treatment choices, and clinical outcomes. Findings from this study emphasized the importance of optimizing the management of the migraine and mental health needs of the patient concurrently and doing so in a manner that is flexible and tailored to the patient’s needs. Patients and physicians strongly believed that improved migraine control would benefit mental health.

Based on these results, patients and physicians appear to be aware of the relationship between migraine and mental health and the importance of treating mental health as part of a comprehensive migraine management plan; however, they may be less aware of the full impact mental health comorbidities have, the barriers faced by patients in accessing mental health services, and how to overcome those barriers. Physicians who treat migraine should proactively solicit and be empathetic to mental health co-morbidities, as well as the internal and external stigmas experienced by these individuals, and actively include them in discussions about their treatment plans.

## Data Availability

Upon request, and subject to review, Pfizer will provide the data that support the findings of this study. Subject to certain criteria, conditions and exceptions, Pfizer may also provide access to the related individual de-identified participant data. See https://www.pfizer.com/science/clinical-trials/trial-data-and-results for more information.
